# Correlation between Disc Imaging Observations and Clinical Efficacy after Percutaneous Endoscopic Lumbar Discectomy: A 1‐Year Follow‐up Study

**DOI:** 10.1111/os.14013

**Published:** 2024-02-21

**Authors:** Bing Li, Tian‐hao Wang, Yi Huang, Yi‐ming Fan, Han Yu, Ao‐qiong Li, Deng‐bin Qi, Qi Wang, Chao Xue, Ze Wang, Guo‐quan Zheng, Yan Wang

**Affiliations:** ^1^ Department of Orthopedics Medical School of the Chinese People's Liberation Army (PLA) Beijing China; ^2^ The First Medical Centre of the Chinese People's Liberation Army (PLA) General Hospital Beijing China; ^3^ Department of Orthopedics The Fourth Medical Center of the Chinese People's Liberation Army (PLA) General Hospital Beijing China; ^4^ Nankai University School of Medicine Nankai University Tianjin China

**Keywords:** Clinical outcomes, Disc morphological changes, Lumbar disc herniation (LDH), Magnetic resonance imaging, Minimally invasive, Percutaneous endoscopic lumbar discectomy (PELD)

## Abstract

**Objective:**

The connection between alterations in the disc structure following percutaneous endoscopic lumbar discectomy (PELD) and symptoms in patients postsurgery has not been reported yet. The purpose of the present study was to discuss the potential correlation between the changes in the morphological characteristics of various reference surfaces of the intervertebral disc after percutaneous endoscopic lumbar discectomy (PELD) and clinical outcomes, to identify the morphological parameters that affect efficacy and provide an evidence‐based foundation for assessing postoperative efficacy.

**Methods:**

From October 2019 to October 2021, after percutaneous endoscopic lumbar discectomy (PELD), 98 individuals were enrolled. MRI DICOM data of the lumbar spine were obtained before and after surgery, specifically around 3 months. The morphological parameters of the operated and adjacent segments of the discs were measured using T2‐weighted images from three reference planes. Outcomes were assessed using the Oswestry disability index (ODI), visual analogue pain scores for the back and leg (VAS‐back/VAS‐leg), Japanese Orthopaedic Association (JOA) scores, and recovery rates. Postoperative changes in disc parameters and outcomes were compared between patients with different severity and types of LDH based on the MSU staging. Patients completed the questionnaire during outpatient follow‐up appointments 3, 6, and 12 months after the surgery. The follow‐up period was 14.69 ± 4.21 months, ranging from 12 to 24 months.

**Results:**

Parameters such as area and circumference of intervertebral discs in the cross‐section were not associated with the change in the efficacy index. Postoperatively, a negative correlation between the variation of the disc height, disc height index, and protrusion distance and the difference in VAS scores for low back pain at 3 and 6 months was observed among the two sagittal change parameters. Differences between changes in disc imaging parameters and postoperative efficacy were not statistically significant between various types of lumbar disc herniation.

**Conclusion:**

For the patients after percutaneous endoscopic lumbar discectomy, the changes in parameters such as disc area and circumference in the cross‐sectional plane are not associated with efficacy, and the changes in disc height and herniation distance in the sagittal plane provide a morphologic basis for the assessment of short‐term postoperative efficacy. In addition, the changes in disc morphologic parameters and postoperative efficacy do not differ between various types of lumbar disc herniation.

## Graphical abstract

Differences in the procedure's effectiveness were not linked to parameter changes in the transverse plane, including the size and perimeter of the discs. The rate of relief of low back pain at 3 months and 6 months after percutaneous endoscopic lumbar discectomy (PELD) was found to be correlated with changes in the disc height and herniation distance in the operated segment in both sagittal planes. After surgery, there were no notable variations in the disc parameters or the effect on the various severity or distinct subcategories of lumbar disc herniation.

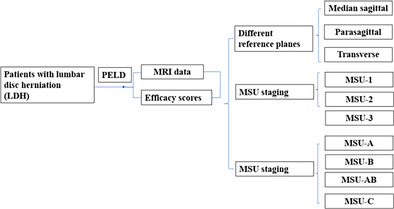

## Introduction

Lumbar disc herniation (LDH) is characterized by a complex clinical presentation that is prone to recurrence and difficult to treat. It manifests as low back pain, limited lumbar mobility, and numbness radiating to the lower extremities.^1^ Due to advancements in spinal endoscopy, percutaneous endoscopic lumbar discectomy (PELD) has emerged as a less invasive treatment option and an alternative to traditional open surgery.^2^ Compared to open surgery, PELD is beneficial in that it can be conducted under local anesthesia as an outpatient procedure, is less invasive, has faster postoperative recovery.^3^, ^4^, ^5^

Most studies have focused on cross‐sectional patient outcome comparisons of conventional *versus* minimally invasive surgery.^6^ The correlation between alterations in the disc structure on images and the surgical results and prognosis pre‐ and post‐minimally invasive surgery has been explored in only a few studies. Thomé *et al*. found that larger disc annular defects and a lower rate of discectomy increased the risk of recurrence of postoperative LDH.^7^ Moreover, the greater the amount of disc volume that is extracted, the more rapid the decrease in disc height in the surgical region after the operation. Research by Heo *et al*. showed no direct correlation between the absolute amount of disc tissue removed during PELD and the postoperative clinical success or postoperative recurrence rates.^8^ Additional studies have also indicated that the actual symptoms are not strongly correlated with the overall size of the herniated disc location and that various factors, including biochemical and psychological factors, play a role in this variation in symptoms. There is a significant difference between these distressing symptoms and the observations on lumbar spine MRI scans.^9^ No specific research has been conducted to clarify the connection between alterations in the disc structure following PELD and symptoms in patients postsurgery.

We will investigate whether there is a correlation between alterations in intervertebral disc imaging scans following PELD and the resulting outcome based on these conducted studies. The aims of this study are as follows: (i) to investigate the potential correlation between changes in morphological characteristics of various disc reference surfaces and clinical outcomes after percutaneous endoscopic lumbar discectomy (PELD); (ii) to identify disc morphological parameters that significantly influence postoperative outcomes; and (iii) to investigate the differences in these parameters and outcomes between patients with different types of lumbar disc herniation (LDH) after surgery. This study helps us to understand further the relationship between morphological changes in lumbar discs and clinical outcomes. It fills a gap in previous studies and provides a scientific basis for optimizing lumbar disc treatment.

## Materials and Methods

### Patient Selection

From October 2019 to October 2021, the clinical data of individuals who underwent PELD treatment for LDH were compiled and examined. This study was registered with the Chinese Clinical Trial Registry under the number ChiCTR2300071767 and was performed in accordance with the Declaration of Helsinki. Written informed consent was obtained from all participants. The clinical data of a total of 119 surgical patients were consecutively recorded throughout this period. To be eligible for inclusion, patients must meet the following criteria: (i) a preoperative diagnosis of LDH, determined based on their clinical presentation, imaging data, and thorough examination of symptoms; (ii) no alleviation of symptoms after 6 weeks of conservative treatment. Based on these criteria, 98 patients were incorporated in this retrospective analysis, and those with multiple‐level surgeries (three patients), loss to follow up (two patients) and previous lumbar surgery (two patients) as well as those not providing sufficient data (14 patients) were excluded from this study. The follow‐up period was 14.69 ± 4.21 months, ranging from 12 to 24 months.

### Surgical Procedures

PELD was performed through either a transforaminal or an interlaminar approach. The level and type of disc herniation and surgeon preference determined the PELD approach.

For patients undergoing PETD, after fluoroscopy on a C‐arm X‐ray device, we routinely disinfected the surgical area, laid out sterile towels, and administered local infiltration anesthesia with 10–20 ml of 1% lidocaine. The target lumbar vertebral segment was punctured with a 16 G puncture needle. After confirming the position of the puncture portion and the puncture needle by fluoroscopy using a C‐arm X‐ray device, a guide wire was inserted, a dilatation catheter was inserted one level at a time, and the bone of the superior articular process was removed with a circular saw to enlarge the intervertebral foramen, a working trocar was created, and, after clarifying its position to the surgeon's satisfaction, a spinal endoscope was inserted to extract the herniated disc tissues meticulously. After ensuring that there was no compression of the nerve roots and that the dural sac was pulsing normally, the endoscope was removed by injecting 1 ml of compound betamethasone and 1 ml of 1% lidocaine into the surgical area. The skin incision was closed with a four‐gauge suture.

For patients undergoing PEID, after fluoroscopy on a C‐arm X‐ray device, we routinely disinfected the surgical area, laid out a sterile towel, and inserted the needle from the lower edge of the prominent side of the intervertebral plate space and the medial inferior articular eminence, and administered local infiltration anesthesia with 10–20 ml of 1% lidocaine. The target lumbar vertebral segment was punctured with a 16 G needle. After fluoroscopy with a C‐arm X‐ray device was used to confirm the position of the puncture portion and the needle, a series of instruments were inserted sequentially, including a guidewire, a dilator, a working channel, and a spinal endoscope. After removing part of the ligamentum flavum, removing fatty tissue, and exposing the nerve roots and dural structures at the lower edge of the spinal canal, the working trocar was rotated, and the dura was pushed toward the contralateral side and protected to extract the herniated disc tissue meticulously. After ensuring that there was no compression of the nerve roots and that the dural sac was pulsing normally, the endoscope was removed by injecting 1 ml of compound betamethasone and 1 ml of 1% lidocaine into the surgical area. The skin incision was closed with a four‐gauge suture.

Postoperatively, patients should have bed rest for about 3 weeks. They were encouraged to perform appropriate rehabilitation exercises as soon as possible after the operation.

### Imaging Parameter Measurements

**FIGURE 1:**
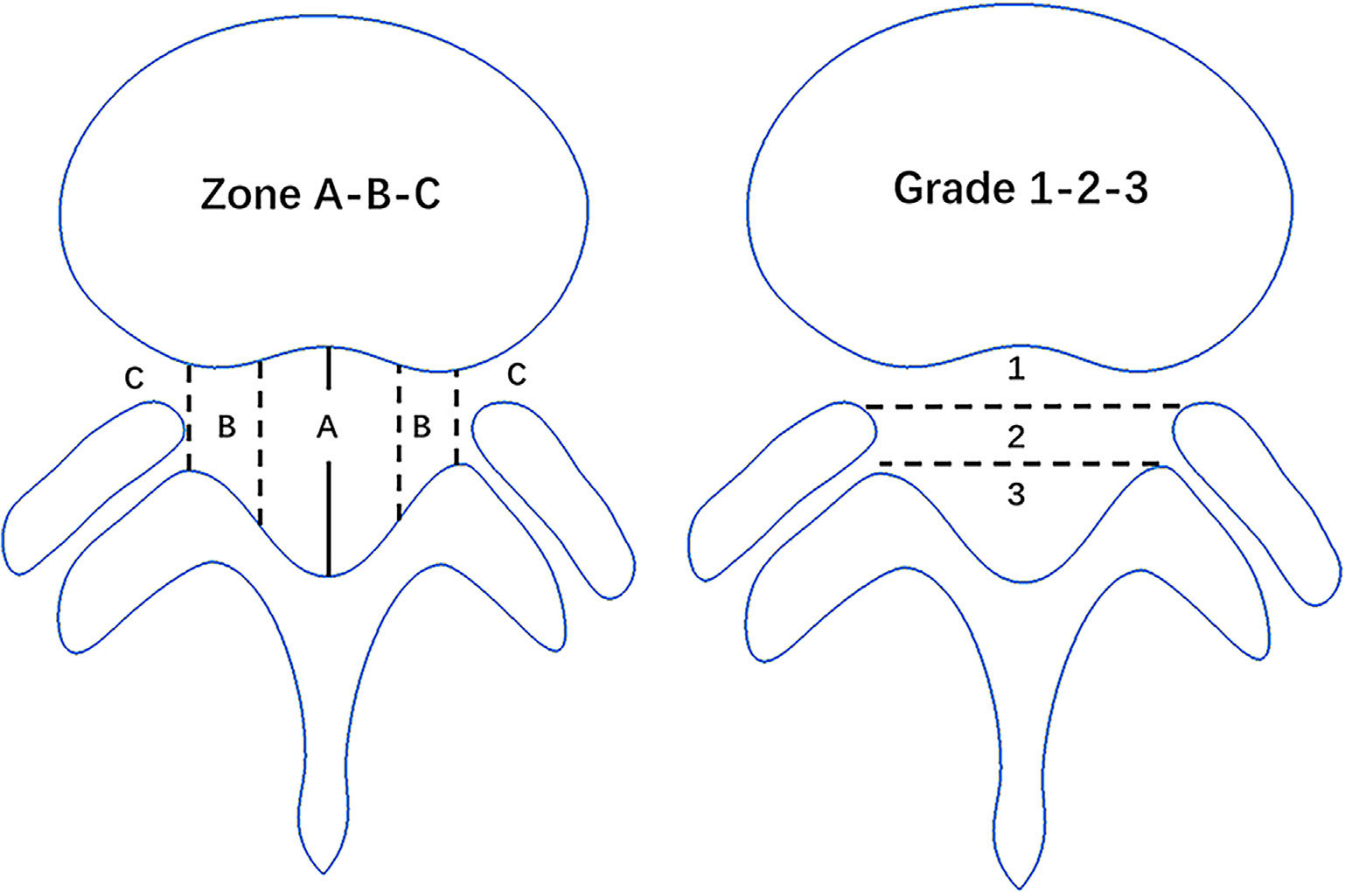
MSU's classification system was used to determine disc herniation grade

**FIGURE 2:**
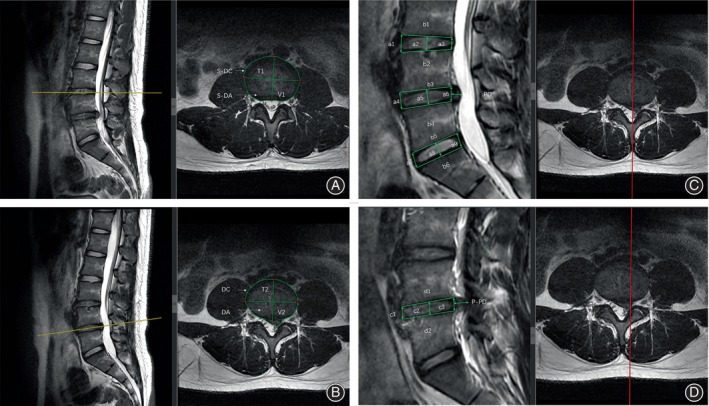
Measurement of MRI parameters. (S‐ = superior segment, P‐ = parasagittal plane, I‐ = inferior segment). (A) superior segment: The vertical diameter (VD, mm), the transverse diameter (TD, mm), the disc circumference (DC, cm), and the disc area (DA, cm^2^). S‐VD=V1, S‐TD = T1, S‐AR = V1/T1. (B) operative segment: The vertical diameter (VD, mm), the transverse diameter (TD, mm), the disc circumference (DC, cm), and the disc area (DA, cm2). VD = V2, TD = T2, AR = V2/T2. (C) Median sagittal plane: The disc height (DH, mm), the disc height index (DHI), the protrusion distance (PD, mm), S‐DH = (a1 + a2 + a3)/3, DH = (a4 + a5 + a6)/3, I‐DH = (a7 + a8 + a9)/3, S‐DHI = (a1 + a3)/(b1 + b2), DHI = (a4 + a6)/(b3 + b4), I‐DHI = (a7 + a9)/(b5 + b6). (D) parasagittal plane: The disc height (DH, mm), the disc height index (DHI), the parasagittal protrusion distance (PD, mm), P‐DH = (c1 + c2 + c3)/3, P‐DHI = (c1 + c3)/(d1 + d2).

The MRI data were obtained from our radiology department's 3.0T MRI scanner (Siemens MAGNETOM Trio, Erlangen, Germany) and exported as DICOM files. Using the patient's lumbar spine MRI data preoperatively and at the first postoperative follow‐up at 3 months, the RadiAnt DICOM viewer (version 2022.1.1) was utilized to measure all the data. From the T2‐weighted images, we acquired the morphological characteristics of the affected disc and the disc of the preceding segment in three reference planes: median sagittal, parasagittal, and transverse. For the parasagittal plane, the image level displaying the most significant protruding disc distance was chosen, while for the cross‐sectional plane, the image level displaying the largest protruding disc area was selected. To ensure data consistency, the image levels chosen from the three reference planes before and after the surgery should be identical for each patient. Specific parameters are defined as follows. The average of the anterior, middle, and posterior disc heights defines disc height (DH) in the sagittal and parasagittal planes.^10^ The disc height index (DHI) is calculated by dividing the sum of the anterior and posterior disc heights by the sum of the superior and inferior disc depths.^11^ The protrusion distance (PD) is the distance from the furthest point of disc protrusion to the height of the posterior intervertebral disc. The vertical diameter (VD) represents the vertical measurement of the disc that passes through the midline of the spinous process. The transverse diameter (TD) is the longest horizontal measurement perpendicular to the vertical diameter. The aspect ratio (AR) is calculated by dividing the vertical diameter by the transverse diameter (VD/TD). The disc circumference (DC) refers to the disc's circumference as it crosses the chosen cross‐section. Finally, the disc area (DA) represents the total area of the intervertebral disc across the selected cross‐sections. Furthermore, we categorized these individuals into three tiers (indicated as 1, 2, and 3) based on severity and four regions (indicated as A, AB, B, and C) based on position following the classification standards for LDH set by Michigan State University (MSU).^12^ (S = superior segment, I = inferior segment, P = parasagittal plane) (Figures 1 and 2).

### Outcome Assessment

The completed questionnaires by the patients are utilized for evaluating the clinical effectiveness. Disability was evaluated using the Oswestry disability index (ODI) questionnaire, pain was scored according to the visual analogue pain scale for the back and leg (VAS‐back/VAS‐leg), and the neurological status of the lumbar spine was evaluated using the Japanese Orthopaedic Association (JOA) score and recovery rate. All patients completed the questionnaire while they were hospitalized before the operation. After the operation, the patients were reviewed in the outpatient clinic at 3, 6, and 12 months, and they completed the questionnaire during each review.

### Statistical Analysis

Categorical variables displayed the count and proportion of cases. The normality of the continuous variables was assessed using the Shapiro–Wilk test. For continuous variables, if they had a normal distribution, they were represented as the means and standard deviations. In contrast, variables without a normal distribution were represented as medians and interquartile ranges. The pre‐and postoperative imaging parameters were analyzed as follows. For paired data that followed a normal distribution, paired samples *t*‐tests were employed, while paired Wilcoxon rank‐sum tests were utilized for data that did not follow a normal distribution. By employing Pearson's correlation coefficient approach, we computed the correlation coefficient. Pearson's correlation coefficient was 0.3, indicating a *p* value below 0.05. Poor evidence of convergent validity is considered for correlations less than 0.3, adequate evidence for correlations 0.3 to 0.6, and very good or excellent evidence for correlations 0.6 or greater.^13^

Here is the intergroup analysis of the various categories of surgical segments: the independent samples *t*‐test is used for continuous variables that are typically distributed, while the Wilcoxon rank‐sum test is used for continuous variables that are not normally distributed. The following is an analysis of the various forms of lumbar disc herniation among different groups. To compare multiple normally distributed groups, we used one‐way analysis of variance (ANOVA) followed by Tukey's post hoc test. For multiple comparisons between groups that were not normally distributed, the Kruskal–Wallis test was employed. Two‐way ANOVA with repeated measures was utilized to compare the various groups at different follow‐up times based on their scores. Measurements were taken twice for all data, with a second measurement taken 3 weeks after the first, and the average of the two results was recorded for analysis. All data were analyzed using R language version 4.2.0 (Lucent Technologies, Murray Hill, NJ, USA), and statistical significance was observed for differences with *p* values less than 0.05.

## Results

### Demographics and Clinical Outcomes

The study population consisted of 31 women and 67 men, with an average age of 44.62 ± 15.59 years. The mean duration of symptoms was 37.96 ± 51.76 months. There was a single case at the L2–3 level, while at the L3–4 level, there were three cases. Additionally, there were 45 cases at the L4–5 level and 49 cases at the L5–S1 level. Furthermore, there were 38, 22, 31, and seven individuals diagnosed with MSU‐A, MSU‐AB, MSU‐B, and MSU‐C variants of LDH, respectively. There were 13, 64, and 21 individuals diagnosed with MSU‐1, MSU‐2, and MSU‐3 forms of LDH, respectively. Percutaneous endoscopic transforaminal discectomy (PETD) was used in 87 cases and percutaneous endoscopic interlaminar discectomy (PEID) in 11 cases. Among the postoperative patients, incisional infection occurred in two cases, nerve root injury in one case, and reprotrusion in five cases. After the surgical procedure, all patients experienced improvements in health outcomes. The ODI, VAS‐B, and VAS‐L scores during postoperative follow‐up were significantly lower than those before surgery, while the JOA scores were significantly higher. There were statistically significant differences between the postoperative and preoperative follow‐up results (*p* < 0.05) (Table 1).

**TABLE 1:** Demographics and clinical outcomes of patients treated with PELD.

Characteristic	Levels	Stats
Total		98 (100.00%)
Age (years)		44.62 ± 15.59
Sex	Male	67 (68.37%)
	Female	31 (31.63%)
Symptom onset (months)		37.96 ± 51.76
Surgical level	L2–L3	1 (1.02%)
	L3–L4	3 (3.06%)
	L4–L5	45 (45.92%)
	L5–S1	49 (50.00%)
MSU (location)	A	38 (38.78%)
	AB	22 (22.45%)
	B	31 (31.63%)
	C	7 (7.14%)
MSU (degree)	1	13 (13.27%)
	2	64 (65.31%)
	3	21 (21.43%)
Methods	PETD	87
	PEID	11
Complications	Infection	2
	Nerve root injury	1
	Reprotrusion	5
ODI (%)	Pre‐op	50.94 ± 12.49
	3M Post‐op	19.94 ± 4.21*
	6M Post‐op	15.45 ± 2.40*
	12M Post‐op	14.71 ± 1.50*
JOA scores	Pre‐op	12.74 ± 3.62
	3M Post‐op	24.97 ± 1.21*
	6M Post‐op	26.31 ± 0.56*
	12M Post‐op	26.76 ± 0.43*
JOA score improvement ratio (%)		85.54 ± 4.76
VAS back pain	Pre‐op	5.32 ± 2.50
	3M Post‐op	1.94 ± 1.07*
	6M Post‐op	1.66 ± 0.82*
	12M Post‐op	1.54 ± 0.86*
VAS leg pain	Pre‐op	6.60 ± 2.53
	3M Post‐op	1.50 ± 0.91*
	6M Post‐op	1.39 ± 0.86*
	12M Post‐op	0.92 ± 1.03*

Abbreviations: 12M, 12 months; 3M, 3 months; 6M, 6 months; JOA scores, Japanese Orthopaedic Association scores; MSU, Michigan State University staging; ODI, Oswestry disability index; Pre‐op, preoperative; Post‐op, postoperative; VAS‐B, visual analogue scale of back; VAS‐L, visual analog scale of leg.

* *p* value is 0.05, compared with pre‐operative.

### Pre‐ and Postoperative Disc Imaging Parameters

Statistically significant imaging parameters postoperatively, compared to preoperatively, included DH, S‐DH, DHI, S‐DHI, PD, P‐DH, P‐DHI, P‐PD, VD, AR, S‐AR, DC, and DA. The postoperative parameters were decreased in comparison to the parameters before the operation (Table 2).

**TABLE 2:** Pre‐ and postoperative disc imaging parameters in patients undergoing PELD.

Parameter	Pre‐op	Post‐op	*p‐*value
DH	10.10 (9.10, 11.50)	9.28 (8.10, 10.50)	0.000**
S‐DH	11.20 ± 1.65	10.96 ± 1.73	0.032*
I‐DH	10.67 ± 1.98	10.38 ± 2.07	0.485
DHI	0.29 (0.30, 0.30)	0.27 (0.20, 0.30)	0.000**
S‐DHI	0.30 ± 0.05	0.29 ± 0.05	0.011*
I‐DHI	0.31 ± 0.06	0.30 ± 0.06	0.295
PD	7.45 (5.70, 9.20)	4.36 (0.00, 5.80)	0.000**
P‐DH	9.82 ± 1.69	8.62 ± 1.87	0.000**
P‐DHI	0.27 ± 0.05	0.23 ± 0.05	0.000**
P‐PD	9.35 (7.80, 10.50)	5.38 (3.00, 6.80)	0.000**
TD	58.80 (56.20, 62.00)	59.05 (55.90, 62.20)	0.640
S‐TD	57.80 ± 4.60	57.90 ± 4.83	0.600
I‐TD	57.49 ± 5.72	57.41 ± 5.62	0.943
VD	44.90 (42.60, 47.70)	42.25 (40.50, 45.10)	0.000**
S‐VD	40.83 ± 3.12	40.61 ± 3.28	0.150
I‐VD	39.68 ± 3.94	39.74 ± 3.97	0.945
AR	0.76 (0.7, 0.8)	0.72 (0.70, 0.80)	0.000**
S‐AR	0.71 (0.70, 0.70)	0.70 (0.70, 0.70)	0.013*
I‐AR	0.69 ± 0.06	0.70 ± 0.06	0.713
DC	16.77 ± 1.31	16.60 ± 1.37	0.016*
S‐DC	16.02 (15.40, 16.90)	16.00 (15.40, 16.90)	0.290
I‐DC	15.59 ± 1.54	15.56 ± 1.58	0.931
DA	21.14 ± 3.11	20.54 ± 3.19	0.000**
S‐DA	19.65 ± 2.94	19.67 ± 2.95	0.820
I‐DA	18.13 ± 3.59	18.12 ± 3.75	0.991

Abbreviations: Pre‐op, preoperative; AR, aspect ratio; DA, disc area; DC, disc circumference; DH, disc height; DHI, disc height index; I‐, inferior segment; P‐, parasagittal plane; PD, protrusion distance; Post‐op, postoperative; S‐, superior segment; TD, transverse diameter; VD, vertical diameter.

* *p* < 0.05;

** *p* < 0.01, pre‐operative *vs*. post‐operative.

### Correlation Analysis

**FIGURE 3:**
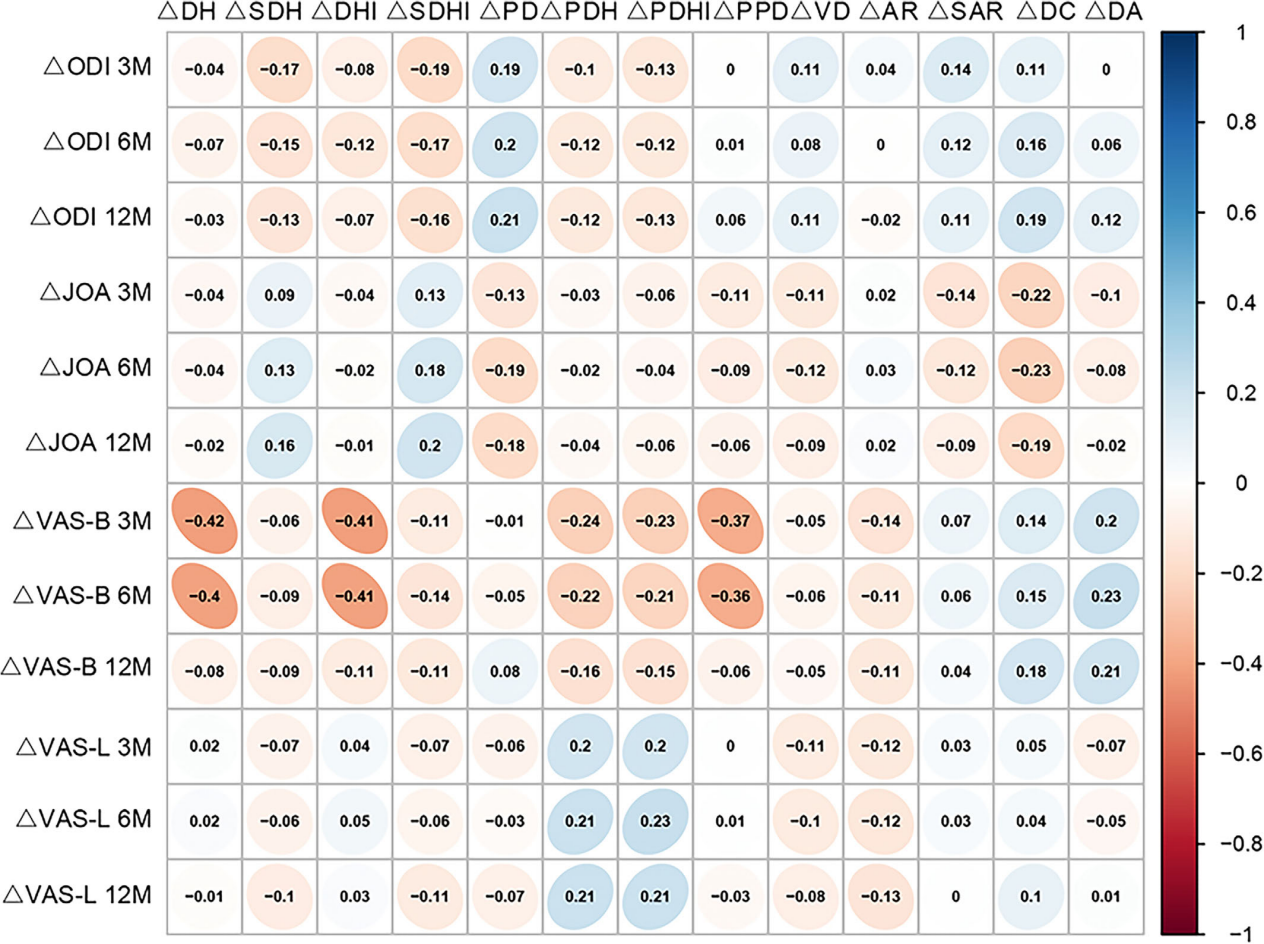
Correlation plot between changes in postoperative imaging parameters and changes in the efficacy indicators.

By employing Pearson correlation analysis, we additionally examined the correlation between the disparity in significant imaging parameters before and after surgery in each reference plane (Δ = preoperative − postoperative) and the magnitude of the alteration in effectiveness indicators during each subsequent postoperative evaluation period. Among the parameters of median sagittal change, the Pearson correlation coefficients indicated a negative correlation between ΔDH and the extent of change in VAS scores for low back pain at 3 months postoperatively and 6 months postoperatively (r = −0.419, *p* < 0.01, and r = −0.402, *p* < 0.01, respectively). The ΔDHI exhibited a negative correlation with the degree of variation in VAS scores for low back pain at 3 months after surgery and at 6 months after surgery (*r* = 0.411, *p* < 0.01 and *r* = 0.409, *p* < 0.01, respectively). The correlation between ΔP‐PD and the change in VAS scores for low back pain at 3 months postoperatively and at 6 months postoperatively was negative (I = 0.367, *p* < 0.01 and *r* = 0.364, *p* < 0.01, respectively) among the parameters of parasagittal change. The differences in parameters of cross‐sectional variation were not correlated with the values of changes in the efficacy indicators (Table 3 and Figure 3).

**TABLE 3:** Correlation coefficients between changes in disc imaging parameters and changes in outcome after PELD.

	△DH	△S‐DH	△DHI	△S‐DHI	△PD	△P‐DH	△P‐DHI	△P‐PD	△VD	△AR	△S‐AR	△DC	△DA
△ODI 3M	−0.043	−0.17	−0.081	−0.185	0.188	−0.103	−0.126	−0.003	0.11	0.044	0.144	0.109	0.004
△ODI 6M	−0.066	−0.148	−0.122	−0.174	0.200*	−0.115	−0.117	0.014	0.081	−0.005	0.119	0.156	0.061
△ODI 12M	−0.033	−0.133	−0.072	−0.162	0.215*	−0.118	−0.128	0.06	0.109	−0.021	0.108	0.191	0.117
△JOA 3M	−0.042	0.088	−0.039	0.13	−0.131	−0.033	−0.06	−0.109	−0.115	0.019	−0.144	−0.217*	−0.098
△JOA 6M	−0.042	0.132	−0.018	0.176	−0.188	−0.022	−0.043	−0.093	−0.116	0.031	−0.124	−0.230*	−0.082
△JOA 12M	−0.021	0.163	−0.011	0.197	−0.18	−0.036	−0.058	−0.061	−0.088	0.02	−0.088	−0.195	−0.019
△VAS‐B 3M	−0.419**	−0.064	−0.411**	−0.11	−0.008	−0.237*	−0.232*	−0.367**	−0.053	−0.14	0.074	0.137	0.200*
△VAS‐B 6M	−0.402**	−0.086	−0.409**	−0.135	−0.054	−0.221*	−0.213*	−0.364**	−0.055	−0.115	0.062	0.153	0.227*
△VAS‐B 12M	−0.079	−0.085	−0.106	−0.115	0.075	−0.155	−0.155	−0.064	−0.045	−0.11	0.036	0.18	0.210*
△VAS‐L 3M	0.02	−0.068	0.042	−0.072	−0.06	0.197	0.200*	0.003	−0.106	−0.119	0.034	0.046	−0.073
△VAS‐L 6M	0.023	−0.061	0.055	−0.06	−0.026	0.213*	0.226*	0.009	−0.102	−0.121	0.034	0.044	−0.048
△VAS‐L 12M	−0.011	−0.095	0.027	−0.106	−0.073	0.210*	0.211*	−0.031	−0.081	−0.134	0.001	0.096	0.012

Abbreviations: 12M, 12 months; 3M, 3 months; 6M, 6 months; AR, aspect ratio; DA, disc area; DC, disc circumference; DH, disc height; DHI, disc height index; JOA scores, Japanese Orthopaedic Association scores; Δ = preoperative − postoperative. ODI, Oswestry disability index; P‐, parasagittal plane; PD, protrusion distance; S‐, superior segment; VAS‐B, visual analogue scale of back; VAS‐L, visual analog scale of leg; VD, vertical diameter.

* *p* < 0.05;

** *p* < 0.01.

### Different Subgroups of Lumbar Disc Herniation

**FIGURE 4:**
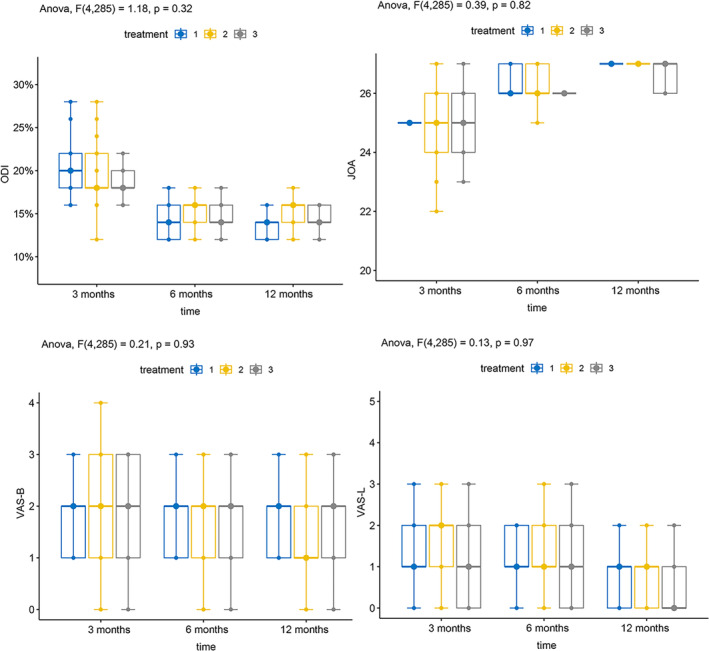
Comparison of changes in disc imaging parameters between the different seize of lumbar disc herniation groups. (A) ODI scores. (B) JOA scores. (C) VAS‐B scores. (D) VAS‐L scores.

**FIGURE 5:**
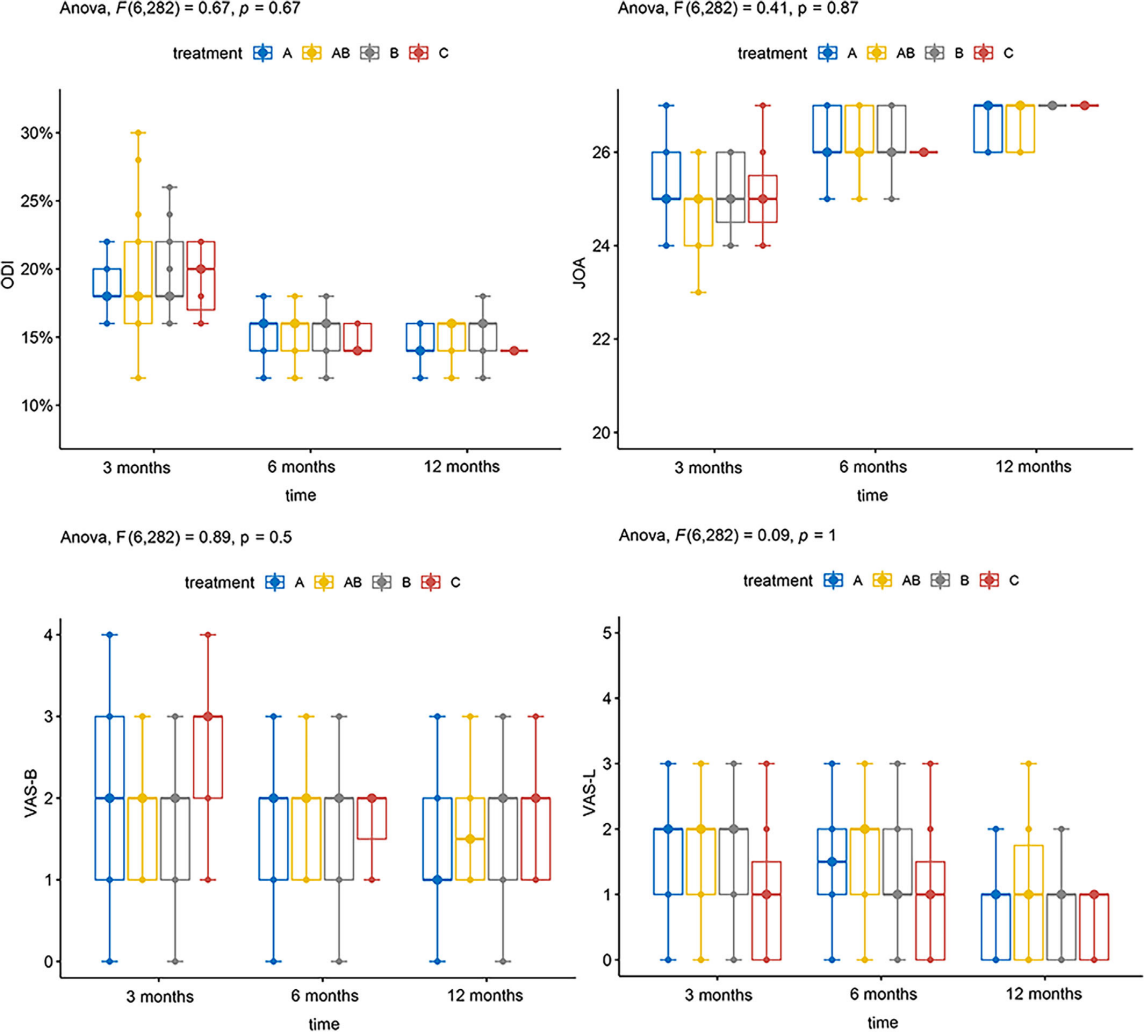
Comparison of changes in disc imaging parameters between the different location of lumbar disc herniation groups. (A) ODI scores. (B) JOA scores. (C) VAS‐B scores. (D) VAS‐L scores.

To examine the variations in imaging parameters and efficacy indicators among the different severity groups (MSU‐1, MSU‐2, MSU‐3) or different types of lumbar disc herniation groups (MSU‐A, MSU‐B, MSU‐AB, and MSU‐C), we conducted a comparison at each time point. At each time point, no notable distinction was observed between the groups concerning alterations in imaging parameters and effectiveness. The figure displays the presented results (Tables 4 and 5, Figures 4 and 5).

**TABLE 4:** Changes in disc imaging parameters in patients with different size of lumbar disc herniation after PELD.

Parameter	1 (N = 13)	2 (N = 64)	3 (N = 21)	*p‐*value
△DH	0.17 (−0.14, 1.62)	1.03 (0.10, 1.78)	1.36 (0.11, 1.99)	0.421
△S‐DH	0.66 ± 1.15	0.16 ± 1.08	0.22 ± 1.04	0.319
△DHI	−0.00 (−0.02, 0.05)	0.03 (0.01,0.06)	0.04 (0.01,0.06)	0.124
△S‐DHI	0.02 ± 0.05	0.01 ± 0.04	0.01 ± 0.04	0.686
△PD	3.03 (1.07, 6.28)	4.09 (1.97, 6.07)	4.26 (2.31, 7.21)	0.551
△P‐DH	0.91 ± 1.43	1.36 ± 1.40	0.88 ± 1.10	0.263
△P‐DHI	0.04 ± 0.04	0.05 ± 0.04	0.03 ± 0.04	0.472
△P‐PD	3.67 (1.83, 4.78)	3.14 (1.96, 6.20)	5.83 (3.08, 7.25)	0.202
△VD	2.40 (0.70, 3.10)	1.55 (1.10, 3.50)	2.70 (1.00, 4.10)	0.849
△AR	0.03 (0.03, 0.06)	0.03 (0.01, 0.05)	0.05 (0.02, 0.08)	0.452
△S‐AR	−0.01 (−0.03, 0.02)	0.01 (−0.01, 0.02)	0.01 (−0.00, 0.03)	0.312
△DC	0.11 (−0.28, 0.74)	0.07 (−0.21, 0.52)	0.29 (0.01, 0.72)	0.168
△DA	1.37 ± 0.60	1.68 ± 0.79	1.57 ± 0.63	0.346

Abbreviations: MSU, Michigan State University staging; AR, aspect ratio; DA, disc area; DC, disc circumference; DH, disc height; DHI, disc height index; P‐, parasagittal plane; PD, protrusion distance; S‐, superior segment; VD, vertical diameter; Δ = preoperative − postoperative.

**TABLE 5:** Changes in disc imaging parameters in patients with different location of lumbar disc herniation after PELD.

Parameter	A (n = 38)	AB (n = 22)	B (n = 31)	C (n = 7)	*p‐*value
△DH	1.09 (0.05, 1.83)	1.44 (0.05, 1.93)	0.97 (0.04, 1.88)	0.25 (0.11, 0.75)	0.658
△S‐DH	0.35 ± 1.09	0.25 ± 1.16	0.13 ± 1.07	0.09 ± 1.08	0.855
△DHI	0.03 (0.01, 0.06)	0.04 (−0.00, 0.06)	0.03 (0.00, 0.07)	0.01 (−0.01, 0.02)	0.235
△S‐DHI	0.01 ± 0.04	0.01 ± 0.04	0.01 ± 0.04	0.01 ± 0.04	0.913
△PD	2.88 (1.19, 7.01)	1.83 (1.00, 5.51)	2.08 (0.62, 4.53)	0.79 (0.61, 3.85)	0.408
△P‐DH	1.20 ± 1.27	1.30 ± 1.16	1.16 ± 1.53	0.94 ± 1.71	0.940
△P‐DHI	0.04 ± 0.04	0.04 ± 0.04	0.04 ± 0.05	0.04 ± 0.06	0.972
△P‐PD	3.70 (1.92, 8.49)	4.45 (2.02, 6.09)	3.64 (2.04, 6.00)	3.20 (1.76, 4.46)	0.792
△VD	2.70 (1.20, 4.10)	1.55 (1.30, 3.20)	1.40 (0.95, 2.50)	1.40 (0.85, 2.10)	0.255
△AR	0.05 (0.01, 0.08)	0.03 (0.01, 0.05)	0.03 (0.02, 0.06)	0.03 (0.01, 0.05)	0.855
△S‐AR	0.01 (−0.02, 0.02)	0.01 (−0.01, 0.03)	0.01 (−0.01, 0.02)	0.02 (0.01, 0.04)	0.528
△DC	0.12 (−0.28, 0.39)	0.08 (−0.19, 0.54)	0.23 (−0.10, 0.62)	0.17 (0.07, 0.48)	0.790
△DA	0.42 (−0.21, 1.25)	0.38 (0.06, 1.11)	0.08 (−0.16, 0.89)	0.62 (0.48, 1.04)	0.104

Abbreviations: AR, aspect ratio; DA, disc area; DC, disc circumference; DH, disc height; DHI, disc height index; MSU, Michigan State University staging; P‐, parasagittal plane; PD, protrusion distance; S‐, superior segment; VD, vertical diameter; Δ = preoperative − postoperative.

## Discussion

We documented the pertinent imaging characteristics in the three reference planes of the treated and neighboring segments on preoperative and postoperative lumbar spine MRI T2 images. Additionally, we assessed the effectiveness of the procedure at various intervals. We found that the change in various parameters on the cross‐sectional plane did not correlate with the change in clinical outcome indicators. However, the postoperative loss of height on the median sagittal plane showed a negative correlation with the decrease in lower back pain scores at 3 and 6 months. Similarly, the decrease in the maximum protrusion distance on the parasagittal plane was negatively correlated with the decrease in lower back pain scores at 3 and 6 months. Following PELD, no disparities were observed in disc imaging parameters and postoperative results in the different severity and herniation type groups.

Numerous studies have proven the efficacy of PELD, indicating that PELD greatly relieved patient symptoms.^5^, ^14^, ^15^ Kang *et al*. indicated that patients who underwent PELD had a significantly higher reoperation rate (5.38%) and a relatively lower infection rate (0.83%) for symptomatic recurrence than those who underwent open lumbar discectomy (OD). However, it is worth noting that OD still had a disadvantage in terms of reoperation rate (2.28%) and infection rate (1.18%) when compared to PELD.^16^ During the follow‐up period, five out of the 98 patients who underwent surgery in our study experienced recurrence and required subsequent surgical intervention, resulting in a recurrence rate of 5.10%.

### Clinical Efficacy and Parameter Variation Are Observed at Three Different Levels

The current clinical research on PELD is on postoperative recurrence and symptom recovery.^17^ Recurrence was not an aim of our current study and needs to be discussed in the future. Our initial aim was to investigate whether there is an association between changes in imaging parameters on disc cross‐section after PELD and postoperative efficacy, and included analysis of other reference surface imaging parameters on this basis.

**FIGURE 6:**
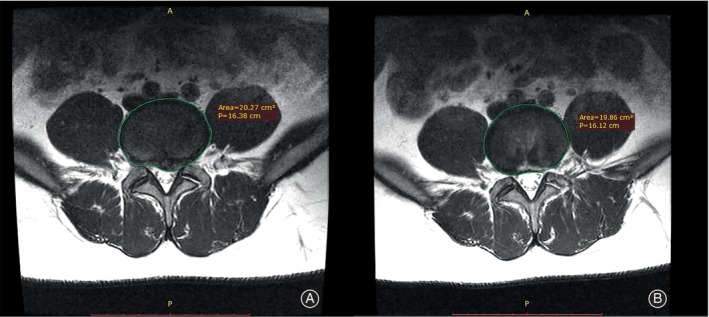
A 29‐year‐old patient is shown preoperatively and postoperatively at the same lumbar spine MRI cross‐section level. The disc area preoperatively was 20.27 cm^2^ and postoperatively was 19.86 cm^2^. The changes in VAS‐B at 3, 6, and 12 months postoperatively were 3, 3, and 3. Those in VAS‐L at 3, 6, and 12 months postoperatively were 8, 9, and 9.

**FIGURE 7:**
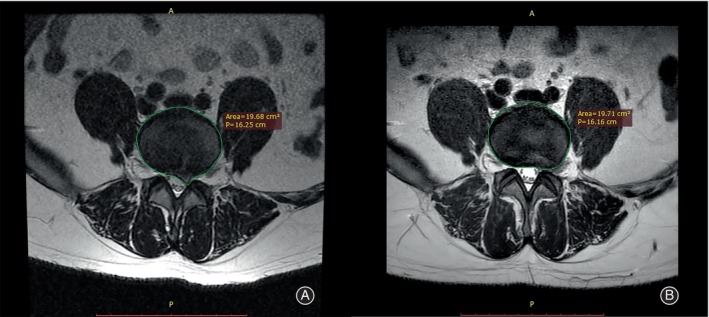
A 39‐year‐old patient is shown preoperatively and postoperatively at the same lumbar spine MRI cross‐section level. The disc area preoperatively was 19.68 cm^2^ and postoperatively was 19.71 cm^2^. The changes in VAS‐B at 3, 6, and 12 months postoperatively were 3, 3, and 4. Those in VAS‐L at 3, 6, and 12 months postoperatively were 6, 6, and 7.

Criteria for decompression in minimally invasive lumbar surgery are indirect confirmation of successful decompression intraoperatively by observation of the free movement of nerve roots or epidural fat, freedom of probe access to the epidural space, and comparison of preoperative magnetic resonance images of the diseased disc with intraoperative removal of disc tissue. However, these criteria are subjective.^18^ Currently, there is limited research on alterations in the disc structure and the related results in individuals following PELD. Heo *et al*. found that absolute disc resection volume was only related to imaging success and not directly related to clinical success or recurrence rates, but that it was possible to calculate postoperative disc residual volume and predict postoperative recurrence rates based on the measurement of the preoperative disc parameters on cross‐sectional MRI, combined with absolute intraoperative resection volume.^8^ According to Heo *et al*.'s findings, it is worth noting that the precise extent of resection during surgery and the imaging parameters after the operation may not always align perfectly, possibly due to the inherent physicochemical characteristics of the disc.^19^, ^20^, ^21^ However, they solely analyzed the correlation between the extent of surgical removal during the operation and the occurrence of recurrence, neglecting to explore the connection between alterations in disc morphology on imaging and measures of effectiveness. In terms of cross‐sectional images, Dunsmuir *et al*. discovered that the size of the herniated discs in conservatively treated patients with LDH did not correlate with pain severity or disability symptoms.^22^ By analyzing the correlation between morphological changes in intervertebral disc cross‐sections before and after imaging studies and the change in efficacy, we discovered that parameters such as absolute area change and aspect ratio on cross‐sections did not directly connect to changes in the final efficacy index. In addition, we have illustrated this occurrence using two instances (Figures 6 and 7). This aligns with the foundation of the earlier research conducted by Dunsmuir.^22^

During our investigation, we noticed a decrease in the height of the operated segment following the surgical procedure and a reduction in the height of the neighboring disc postsurgery. We observed a similar decline in the average disc height and the height index. Before surgery, the segmental DH measurement was 10.10 (9.10, 11.50), while the DHI measurement was 0.29 (0.30, 0.30). The segmental DH measurement before surgery was 11.20 ± 1.65, with a DHI measurement of 0.30 ± 0.05. After surgery, the DH of the specific segment was measured to be 9.28 (8.10, 10.50), with a DHI of 0.27 (0.20, 0.30). Before surgery, the DH of the same segment was recorded as 10.96 ± 1.73, along with a DHI of 0.29 ± 0.05. McGirt and colleagues in a prospective cohort study involving 108 cases found that the disc height in the operated segment decreases over time following discectomy. After surgery, the height of the segmental discs that were surgically treated decreases by 18% at 3 months and by 26% at 2 years. Furthermore, as the amount of the disc that is extracted increases, the speed at which the disc height diminishes accelerates.^23^ The correlation between the change in protrusion distance (ΔP‐PD) and the extent of disc removal is significant, as the extent of removal also impacts the subsequent decrease in disc height after surgery. Our research revealed a negative correlation between decreased lower back pain scores 3 and 6 months after surgery and ∆DH and ∆DHI in the median sagittal plane. Additionally, we observed a negative association between the reduction in lower back pain scores at 3 and 6 months postoperatively and ∆P‐PD in the parasagittal plane. The decrease in VAS‐B scores at 3 and 6 months can be smaller when there is a higher value of disc height loss in the operated segment and a more significant reduction in herniation distance after surgery. Our findings suggest that the rate of alleviation of lower back pain within 6 months after surgery may be influenced by the extent of disc height reduction and the magnitude of protrusion distance alteration in the sagittal plane of the treated area. Regarding this phenomenon, certain studies have indicated that the reduction in disc height is frequently accompanied by alterations such as diminished pressure within the disc and heightened stress on the adjacent joints due to dehydration. Consequently, this results in increases in postoperative pain.^24^ Wang *et al*. revealed that the collapse of disc height after transforaminal endoscopic lumbar discectomy was linked to short‐ and long‐term postoperative low back pain in a multicenter study.^25^ Yet there are some different views. According to the research conducted by Kursumovic *et al*., it is proposed that a decrease in DH could potentially result in a decrease in the movement between the vertebrae, consequently alleviating the discomfort linked to the motion of the lumbar spine.^26^ Currently, research clarifying the relationship between PELD and postoperative low back pain is still lacking. Zhong and colleagues found that prior to surgery, Modic alterations, accumulation of fat in the muscles surrounding the spine, and inflammation of the lumbodorsal fascia were notable elements contributing to the persistence of low back pain after the operation.^27^ For most patients treated with PELD surgery, approximately 15–25% of individuals still experience lower back pain after surgery. Persistent lower back pain after surgery may be linked to loss of disc height, extent of re‐herniation, quality of trephine/cannula position, and sex.^25^ Future studies are needed to elucidate the causes that affect low back pain after PELD.

### Various Forms of Lumbar Disc Herniation

PELD treats almost all types of LDH.^28^, ^29^, ^30^, ^31^ The authors Dewin and others found that among a younger group of patients, the outlook for patients who underwent surgery for occult or herniated lumbar disc herniation was significantly better than for those with contained lumbar disc herniation.^32^ However, the criteria for classification in their research were not concrete enough. Therefore, a more explicit MSU staging based on herniation severity and location of herniation was used in our analyses. Variances in findings between studies may arise due to the utilization of diverse classification criteria. Future large‐scale multicenter and prospective studies are necessary for a more precise conclusion. No significant differences in morphological parameter changes were observed in the three postoperative reference surfaces among the various types of lumbar disc herniation in the patient population under our study. Additionally, postoperative outcomes did not show any significant variations among the various types of lumbar disc herniation.

### Limitation and Strengths

Our study has various additional constraints. First, this study was retrospective, and there may be some bias and confounding factors. This study recorded consecutive surgical patients to minimize selection bias, but type II errors are possible. Furthermore, we exclusively gathered lumbar spine MRI information during the patient's initial evaluation approximately 3 months after the surgery, and it is possible that the structural alterations in the disc after the operation could be subject to change over time. For future studies, it would be beneficial to document the lumbar spine MRI data during each evaluation period and establish a correlation with the corresponding efficacy indicators.

In this study, we demonstrated innovation and rigor. On the one hand, we delved into the correlation between disc imaging changes and clinical outcomes after PELD, which filled the gap of previous studies. On the other hand, we included a more significant number of cases and used multidimensional measurements, which ensured the reliability and accuracy of the study results and laid a solid foundation for further exploration.

## Conclusions

The changes in parameters such as disc area and circumference in the cross‐section did not affect the efficacy, while the speed of low back pain relief in 6 months after PELD surgery was correlated with changes in the disc height and the protrusion distance in the sagittal plane. Furthermore, there were no notable variances in disc parameter changes and curative effect between different types of lumbar disc herniation after surgery.

## Declaration

All authors listed meet the authorship criteria according to the latest guidelines of the International Committee of Medical Journal Editors, and all authors read and approved the final manuscript.

## Author Contributions

All authors contributed to the study conception and design. Bing Li, Tian‐hao Wang: conceptualization, methodology, formal analysis, writing—original draft, writing—review and editing, project administration. Yi Huang, Yi‐ming Fan and Han Yu: formal analysis, investigation, supervision. Ao‐qiong Li, Deng‐bin Qi, Qi Wang, Chao Xue and Ze Wang: investigation, visualization, resources, validation, data curation. Guo‐quan Zheng and Yan Wang: conceptualization, supervision, project administration. Guo‐quan Zheng and Yan Wang should be considered joint corresponding authors. Bing Li and Tian‐hao Wang should be considered joint first authors.

## Ethics Statement

This study was registered with the Chinese Clinical Trial Registry under the number ChiCTR2300071767 and was performed in accordance with the Declaration of Helsinki. Written informed consent was obtained from all participants.

## Conflict of Interest Statement

On behalf of all authors, the corresponding author states that there is no conflict of interest.
